# Using robotic technology to quantify neurological deficits among survivors of critical illness: do they relate to brain tissue oxygen levels? a pilot study

**DOI:** 10.1186/2197-425X-3-S1-A730

**Published:** 2015-10-01

**Authors:** M Wood, D Maslove, J Muscedere, S Scott, JG Boyd

**Affiliations:** Queen's University, Centre for Neuroscience Studies, Kingston, Canada; Queen's University, Critical Care, Kingston, Canada; Queen's University, Critical Care/Medicine, Kingston, Canada; Centre for Neuroscience Studies, Kingston, Canada

## Introduction

Long-term cognitive dysfunction is common among survivors of critical illness. The etiology of this cognitive dysfunction is unknown, but it may relate to cerebral hypoxemia and hypoperfusion. Near infrared spectroscopy (NIRS) has been used to measure brain tissue oxygenation(BtO2) in patients during cardiac surgery and after cardiac arrest. Preliminary studies have suggested that BtO2 levels may correlate with neurological recovery.

The KINARM robot provides quantitative metrics of sensory, motor, and cognitive function involving the upper limbs. It can quantify sensory processing of the limb, basic motor skills as well as a range of cognitive processes including executive function, working memory, and attention. There is a large normative database for the majority of tasks to which patient performance can be compared. It can detect subtle neurological deficits post ischemic stroke, which are not apparent on routine clinical testing. It is unknown if deficits can be identified in survivors of critical illness.

## Objective

The overall OBJECTIVE of our research program is to explore the relationship been BtO2 levels and quantitative metrics of neurological dysfunction as measured by the KINARM. The AIM of this pilot study was to assess the feasibility of our study protocol.

## Methods

A prospective single centre observational study performed at a tertiary level medical surgical intensive care unit (ICU). Adult patients were enrolled if they were unplanned admissions to the ICU, and required either ventilation for >24 hours, and/or vasoactive agents. They were excluded if they had a prior history of dementia or their life expectancy was< 3 months. BtO2 was measured with the FORESIGHT NIRS monitor for the first 24 hours of ICU admission. Patients were assessed 3 months after ICU admission with the KINARM.

## Results

From February 2014-April 2015 26 patients have been enrolled. Five of these patients have been assessed with the KINARM robot. Of the remaining 21 patients, 17 died, and 4 refused follow up assessment.. All patients assessed performed within normal limits on simple sensorimotor tasks, such asproprioception and visually guided reaching. However, 4/5 patients scored outside the normal range when the task became more complicated by inverting the patient response by 180 degrees. On a task of interlimb coordination (“ball on bar”) task, as well as a robotic version of the Trails A and Trails B test, only one patient scored below the normal range. Interestingly, this patient had the lowest mean BtO2.

## Conclusions

It is feasible to use the KINARM robot to identify neurological deficits after critical illness. This pilot study has established the infrastructure for our larger, observational study examining the relationship between physiological variables during critical illness (including BtO2) and quantitative metrics of neurological recovery.

